# Assessing the impact of oscillating dietary crude protein on the stability of the rumen microbiome in dairy cattle

**DOI:** 10.3389/fmicb.2025.1568112

**Published:** 2025-06-10

**Authors:** F. L. Viquez-Umana, M. G. Erickson, J. D. Young, G. I. Zanton, M. A. Wattiaux, G. Suen, H. C. Mantovani

**Affiliations:** ^1^Department of Animal and Dairy Science, University of Wisconsin – Madison, Madison, WI, United States; ^2^USDA-ARS, U. S. Dairy Forage Research Center, Madison, WI, United States; ^3^Department of Bacteriology, University of Wisconsin – Madison, Madison, WI, United States

**Keywords:** rumen microbiome, microbial diversity, dietary crude protein, feeding pattern, oscillating protein diet

## Abstract

**Introduction:**

Understanding how the rumen microbiota responds to varying protein levels and feeding patterns is critical for optimizing dairy cattle nutrition. This study investigated the influence of dietary crude protein (CP) levels (13.8% or 15.5% CP of ration dry matter) and CP feeding patterns (constant over time (static) or oscillating by 1.8 percentage units above and below the mean every 48 h) on the composition, diversity, and function of the rumen microbiome.

**Methods:**

Using a replicated Latin Square design, eight rumen-cannulated Holstein cows were assigned each of the four dietary treatments (structured as a 2 × 2 factorial) in four consecutive 28-day periods (with 24 days of adaptation and 4 days of sampling). Rumen samples were collected 4 h post-feeding, and amplicon libraries of the V4 region of the 16S rRNA gene were sequenced and analyzed to assess changes in microbiome composition. Additionally, volatile fatty acids (VFAs) were measured to evaluate rumen microbial function.

**Results:**

Results indicated that dietary CP level did not alter microbial diversity (*p* = 0.30), but oscillating diets increased rumen microbial diversity (Shannon index, *p* = 0.04). The rumen microbiome richness was also affected by CP feeding pattern (*p* = 0.05), but not dietary CP level (*p* = 0.27). Furthermore, differential abundance analysis using ANCOM-BC identified CAG- 352 (*p* = 0.0001) and an unclassified member of the family Acholeplasmataceae (*p* = 0.0002) as taxa significantly impacted by protein level and feeding pattern, even though their relative abundance was low (below 0.02%). The functional profile of the rumen bacterial communities was not affected by CP level or feeding pattern, and VFA profiles also remained consistent across treatments, with no observable changes in concentration.

**Discussion:**

These findings support the hypothesis that the rumen microbiome remains stable despite variations in the ruminal supply of dietary CP, suggesting that compensatory mechanisms may be involved. Although oscillating dietary CP concentration might alter the rumen microbiome, further research into rumen metabolic processes and host-microbiome interactions is needed to evaluate if the changes observed in our study are biologically relevant for developing new opportunities to enhance protein nutrition in dairy cattle.

## 1 Introduction

The symbiotic relationship between mammals and their associated microbiota is essential for maintaining host health and function, and these interactions are particularly prominent in ruminants (Lin et al., 2019). Ruminants are herbivores with a pre-gastric compartment - the rumen - specialized in microbial fiber degradation (Russell and Rychlik, 2001). The rumen microbiome, comprised of bacteria, archaea, protozoa, viruses, and fungi (Cammack et al., 2018), plays an important role in maintenance, growth, reproduction, and milk production by fermenting feed into volatile fatty acids (VFAs) that are used by the host as an energy source and building blocks of body tissues and milk components (Jami et al., 2013; Liu et al., 2021). In addition, rumen microbes synthesize amino acids and vitamins that contribute to overall animal health (Cammack et al., 2018). Importantly, microbial crude protein is the primary source of amino acids for ruminants, making it a key factor in their nutrition (Russell and Rychlik, 2001).

The production of microbial crude protein and the metabolism of nitrogen (N) within the rumen are complex processes. With adequate carbohydrate and N supply, microbial biomass increases, leading to enhanced microbial protein production (Firkins et al., 2007). N metabolism in the rumen involves the degradation of dietary amino acids into ammonia, which can be utilized by other microbes as a N source or absorbed into the ruminant’s portal vein (Tan et al., 2021). Once absorbed, N from ammonia is readily converted to urea, which can be recycled back into the gastrointestinal tract or irreversibly lost as urinary urea-N or metabolic fecal N (Lapierre and Lobley, 2001).

Urinary urea-N poses environmental concerns, as it can leach into aquifers and contribute to increased levels of nitrate in drinking water. It can also contribute to acidification of surface waters and algal blooms, compromising both human and ecosystem health (Erisman et al., 2013). Consequently, various strategies have been explored to reduce N excretions from the dairy sector, such as lowering the supply of degradable protein (Edouard et al., 2016), oscillating protein content of diets (Rauch et al., 2021; Erickson et al., 2023), and employing selective breeding strategies (Beatson et al., 2019).

We previously reported that dairy cattle maintained milk and component production with both static and oscillating dietary protein supplies, regardless of whether the protein content was high or low (Erickson et al., 2023). These findings suggested that dairy cows rely on still poorly understood adaptation mechanisms in response to daily variations in dietary crude protein (CP) supply. However, while production outcomes have been well-documented, there is a significant gap in our understanding of how such dietary variations impact the stability of the rumen microbial ecosystem (Parra et al., 2022).

Given the role of the rumen microbiome in fermentation and protein synthesis, we hypothesized that oscillating dietary CP levels could induce changes in microbial diversity and composition, potentially affecting the functional capacity of the rumen microbiota. Thus, we explored whether oscillating protein levels destabilized the microbial community, or alternatively, whether the microbiome exhibited resilience, maintaining stability through compensatory mechanisms. Understanding these responses is crucial for optimizing dietary strategies that balance production efficiency with environmental sustainability.

To address this knowledge gap, our study aimed to analyze the effects of varying protein content and feeding patterns (oscillating vs. static) on the rumen microbiome of lactating dairy cows. Specifically, we investigated the composition, diversity, and functional output of the rumen microbial community under these dietary conditions, with a particular focus on how substrate availability influences the structure and function of the microbiome.

## 2 Materials and methods

### 2.1 Study design and sample collection

This study was part of a larger study, with productivity data previously described by Erickson et al. (2023) and nutrient digestibility, nitrogen balance, and plasma amino acid results reported in Erickson et al. (2024). In brief, the study used a 2×2 factorial arrangement of treatments with a replicated Latin Square design. For the current analysis of ruminal parameters, we used a subset of multiparous lactating Holstein cows (*n* = 8; with a mean of 135 ± 9 days in milk) fitted with 10 cm ruminal cannulas. Each 28-day experimental period included 24 days of diet adaptation followed by a 4-day sampling period. For the oscillating condition, the 4-day sampling period included a higher-CP phase (2 days) and a lower-CP phase (2 days; Supplementary Figure 1). Due to the occurrence of mastitis, two cows were removed from the study during period 2, and one cow was removed during period 4. A replacement cow was introduced in period 3 to maintain the sample size.

Rumen samples were collected separately for microbial analysis and rumen fluid chemical composition on days 25, 26, 27, and 28 of each experimental period, representing 2 days of higher-CP and 2 days of lower-CP feeding for the oscillating condition. For microbial analysis, rumen contents were collected manually at 1200 h (approximately 4 h post-feeding) from three distinct locations across the medial ventral rumen, composited by approximately equal volumes, filtered through four layers of cheesecloth to separate solids from fluid, and then solid and fluid samples were immediately flash-frozen in liquid N before being stored at −80°C. For rumen fluid chemical composition analysis, rumen fluid samples were obtained from the ventral rumen at 0600, 0800, 1000, and 1900 h using a metal probe inserted through the cannula, and these were stored at −20°C for subsequent analysis of VFA and ammonia-N concentrations.

### 2.2 Dietary treatments

Ingredient and nutrient composition of dietary treatments were reported in Erickson et al. (2023). The dietary treatments evaluated the effects of two factors: dietary CP level (high vs. low) and dietary CP feeding pattern (oscillating vs. static). Diets were formulated according to NRC (2001) guidelines, with the high dietary CP condition (mean = 15.5, SD = 0.8% of DM) predicted to slightly exceed rumen degradable and metabolizable protein recommended supplies, and the low dietary CP condition (mean = 13.8, SD = 0.6% of DM) predicted to result in a deficiency of rumen degradable and metabolizable protein (Erickson et al., 2023). We tested static and oscillating patterns of dietary CP feeding. In the static feeding pattern, cows received a consistent CP level (high or low) throughout each experimental period. In the oscillating pattern, the CP level changed every 48 h to 1.8% [DM basis] above or below the corresponding CP concentration (i.e., high = 15.5% ± 1.8% CP or low = 13.8% ± 1.8% of CP) throughout the experimental periods. The diets maintained a constant forage-to-concentrate ratio of 60:40, with adjustments in soybean meal, ground corn, expeller soybean meal, and soybean hulls to achieve the target CP levels while keeping forage (corn silage and alfalfa haylage) concentration, neutral detergent fiber:starch, and rumen degradable protein: CP ratios consistent (Erickson et al., 2023).

### 2.3 Sample processing

#### 2.3.1 DNA extraction

DNA from the samples was extracted with a modification of the PCSA method (phenol:chloroform with bead beating II), as previously described (Henderson et al., 2013). First, the filtered rumen contents stored at −80°C were thawed at 4°C for 24 h. Then, the liquid samples were centrifuged for 30 min at 8,000 g, and the supernatant was discarded. Both solid and liquid samples were mixed with extraction buffer (Tris–HCl 0.1 M, EDTA 0.01 M, and NaCl 0.15 M). The extraction buffer was used as a negative control for the DNA extraction. Duplicates of 1 mL of the resuspended sample were transferred into a microcentrifuge tube with 700 μL equilibrated phenol (Sigma-Aldrich, St. Luis, MO), 50 μL of 20% sodium dodecyl sulfate (Sigma-Aldrich, St. Luis, MO), and 0.5 g of zirconium/silica beads (0.1 mm diameter, BioSpec Products, Bartlesville, OK). Cell lysis was performed by bead beating, twice for 2 min, with an incubation of 10 min at 60°C between bead beating cycles. These tubes were then centrifuged at 12,000 g for 10 min, and the DNA was recovered from 850 μL of the supernatant. Briefly, DNA was recovered by consecutively vortexing the supernatant with equal volumes of phenol-chloroform-isoamyl alcohol [PCI, 25:24:1 (vol/vol/vol)], followed by a 10-min centrifugation at 12,000 g. The DNA was precipitated by gently mixing 500 μL supernatant with 50 μL 2 M sodium acetate and 300 μL of isopropyl alcohol. This mixture was incubated for 2 h or overnight at −20°C, followed by centrifugation at 12,000 g for 20 min at 4°C. Finally, the pellet was washed with 1 mL of ice-cold 70% ethanol and dried for 90 min or overnight prior to resuspension in elution buffer (10 mM Tris and 1 mM EDTA).

#### 2.3.2 Library reconstruction and DNA sequencing

DNA concentration was measured using the Qubit broad-range kit on an Invitrogen Qubit fluorometer (Thermo Fisher Scientific, Waltham, MA), and samples were amplified by PCR targeting the V4 region of the 16S rRNA gene using universal bacterial primers (515F: GTGCCAGCMGCCGCGGTAA and 806R: GGACTACHVGGGTWTCTAAT) with distinct barcodes and Illumina MiSeq sequencing adapters (Kozich et al., 2013). A total of five PCR reactions were conducted, each with a negative control. Amplicons were separated from residual primers and genomic DNA via gel electrophoresis (1% low-melt agarose) and extracted and purified using a Zymoclean Gel DNA Recovery Kit (Zymo Research, Irvine, CA). The purified amplicons of each sample were pooled across solid and fluid fractions at an equimolar concentration of 4 nM, with final concentrations verified using the Qubit High Sensitivity kit. Sequencing was performed on an Illumina MiSeq sequencer using a 250 bp paired-end sequencing kit, with 10% PhiX control and a final loading concentration of 10 pM.

#### 2.3.3 VFA, pH, ammonia, and total amino acids quantification

Rumen fluid samples were thawed at 4°C and analyzed for VFAs using gas chromatography (GC-2010 Plus, Shimadzu Scientific Instruments). Due to unreliable standards, valerate results were excluded from the analysis. Ruminal pH was measured within 20 min of the sample collection using a portable pH-meter (WTW 3110 meter; Xylem, Rye Brook, NY). Ruminal ammonia levels were measured using the phenol/hypochlorite method, and total amino acids were standardized to leucine (Method 18–218-00-X; Lachat) on a Lachat Quik-Chem 8,000 flow injection analysis system (Lachat Instruments).

### 2.4 Microbiome bioinformatic analysis

First, the amplicon sequences were processed using the QIIME2 platform v2023.9 (Bolyen et al., 2019). Sequences were clustered into amplicon sequence variants (ASVs) using the DADA2 algorithm and the “dada2 denoised-paired” plugin (Callahan et al., 2016), with forward reads trimmed at base 249 and reverse reads at base 242 to remove low-quality bases. The resulting ASVs were aligned with MAFF (Katoh et al., 2019), and a phylogenetic tree was constructed using FastTree (Price et al., 2010) via the “phylogeny align-to-tree-mafft-fasttree” plugin. Taxonomy assignment was performed using a Naive Bayes classifier trained on the Silva 138 database, trimmed to the V4 region of the 16S rRNA gene (Robeson et al., 2020).

Then, the ASV table, phylogenetic tree, and taxonomy classification were imported into the phyloseq R package (McMurdie and Holmes, 2013). ASVs classified as contaminants based on their prevalence in DNA extraction and against PCR controls were identified and removed using the decontam R package (Davis et al., 2018). Additionally, ASVs corresponding to unassigned domains, chloroplast sequences, or mitochondrial sequences were also excluded.

For diversity analysis, samples were rarefied to 12,900 sequences. This rarefaction depth was selected based on the samples’ rarefaction curves (Supplementary Figure 2). The Shannon diversity index and ASV richness were calculated using phyloseq. Faith’s phylogenetic diversity index was estimated with the R package btools (Battaglia, 2023). Alpha diversity indices (inverse Shannon’s and ASV richness) were visualized using ggplot2 (Wickham, 2016). To compare the composition of the samples, a beta diversity analysis was performed using weighted UNIFRAC (Lozupone et al., 2011) and Bray and Curtis (1957) distance matrices, and principal coordinates analysis (PCoA) plots were generated using phyloseq.

To evaluate the functional profile of the rumen bacterial communities in response to experimental diets, we employed Phylogenetic Investigation of Communities by Reconstruction of Unobserved States (PICRUSt2, v2.5.2) that maps 16S rRNA sequences to reference genomes and gene families to predict the functional profile of bacterial communities (Douglas et al., 2020). Briefly, PICRUSt2 analysis was performed using our 16S rRNA gene sequencing data (ASV table in biom-format) as input, and predictions were made for EC numbers (Enzyme Commission number) and their respective KO identifier/functional ortholog (KO) that corresponds to a pathway node or a hierarchy node in the Kyoto Encyclopedia of Genes and Genomes database (Kanehisa and Goto, 2000). The resulting table was exported to R and filtered for selection of predicted KOs related to protein metabolism pathways (e.g., nitrogen, sulfur, nucleotide, and amino acid metabolism). Then, the filtered KO table was used for exploratory and statistical analyses.

The microbial communities were analyzed through a series of comparative approaches reflecting the 2 × 2 factorial treatment arrangement. Initially, the main effects of feeding pattern (oscillating vs. static) and protein level (high vs. low) were compared across all samples to assess overall differences. The data were then stratified into four distinct subsets: (1) oscillating feeding pattern with high dietary CP, (2) oscillating feeding pattern with low dietary CP, (3) static feeding pattern with high dietary CP, and (4) static feeding pattern with low dietary CP. Then, pairwise comparisons were made within these subsets. Also, dietary CP levels were compared within each feeding pattern, and feeding patterns were compared within each dietary CP level. Finally, the interaction between feeding pattern and dietary CP level was assessed by comparing the microbial communities across all four dietary groups to assess the combined effects on microbial community composition.

### 2.5 Statistical analysis

Statistical analyses were conducted in R version 4.2.2, with *p* < 0.05 considered significant and 0.05 ≤ *p* ≤ 0.10 considered tendencies. Nine linear models were tested for each alpha diversity index, with the best model selected based on Akaike Information Criterion (AIC) values and weights (see Supplementary Table 1). Additionally, two mixed linear models fitted with the R package nlme (Pinheiro et al., 2024) were tested with the period and cow considered as random variables. In the first mixed linear model, we included the effect of dietary CP feeding patterns and dietary CP levels, while in the second mixed linear model, the interaction between patterns and levels was also included. However, none of the mixed linear models detected significant differences in alpha diversity indexes across treatments (*p* > 0.05) and were not included in the downstream analyses.

The models (linear model 1 and 7, Supplementary Table 1) that captured significant differences in alpha diversity indexes based on the dietary CP feeding pattern or its interaction with dietary CP level included dietary CP level and feeding pattern as fixed effects and were selected for the graphical representations to account for any possible differences in alpha diversity across treatments. Normality was assessed using the Shapiro–Wilk test.

For VFA and ammonia-N, new linear models that included sampling time were tested, and the best model was selected based on ACI values and weights. Given that the residuals did not meet normality assumptions, non-parametric Kruskal-Wallis tests were applied. For beta diversity, permutational multivariate analysis of variance (PERMANOVA) was conducted using the adonis2 function from the vegan R package (Oksanen et al., 2022). The differential abundance of taxa was evaluated using ANCOM-BC2 (Lin and Peddada, 2024), with *p*-values adjusted using the false discovery rate (FDR) method.

We applied a linear regression model (LinDA; Zhou et al., 2022) on the centered log-ratio transformed KO table predicted by PICRUSt2 to evaluate the predicted bacterial functional profile differentially abundant. Besides accommodating mixed effects, this model corrects bias due to the compositional effect of sequencing data and controls for false discovery rate (FDR) (Zhou et al., 2022). In our model, cow and period were designated as random effects, while dietary CP level, CP feeding pattern, and their interactions were treated as fixed effects. Log2FoldChange values were estimated only for predicted KOs associated with protein metabolism pathways and were present in at least 80% of our samples. Differences were considered statistically significant when FDR-adjusted *p*-values were ≤ 0.05.

## 3 Results

### 3.1 VFA profile, ammonia-N

**Figure 1:**
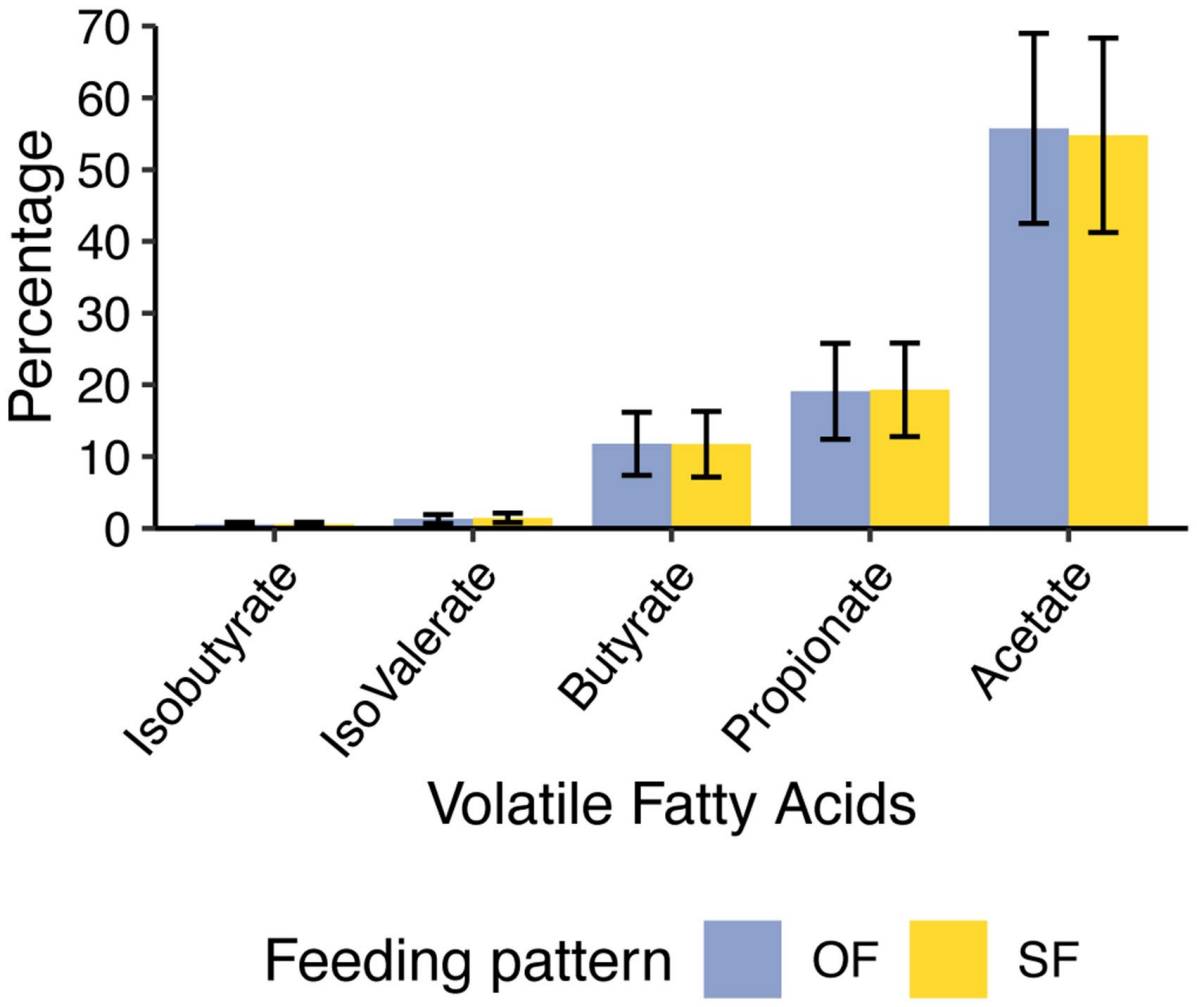
Profile of ruminal volatile fatty acids (VFAs) concentration under oscillating feeding (OF) or static feeding (SF) patterns. Error bars represent the mean percentage of each VFA detected ± the standard deviation for each feeding pattern.

**Figure 2:**
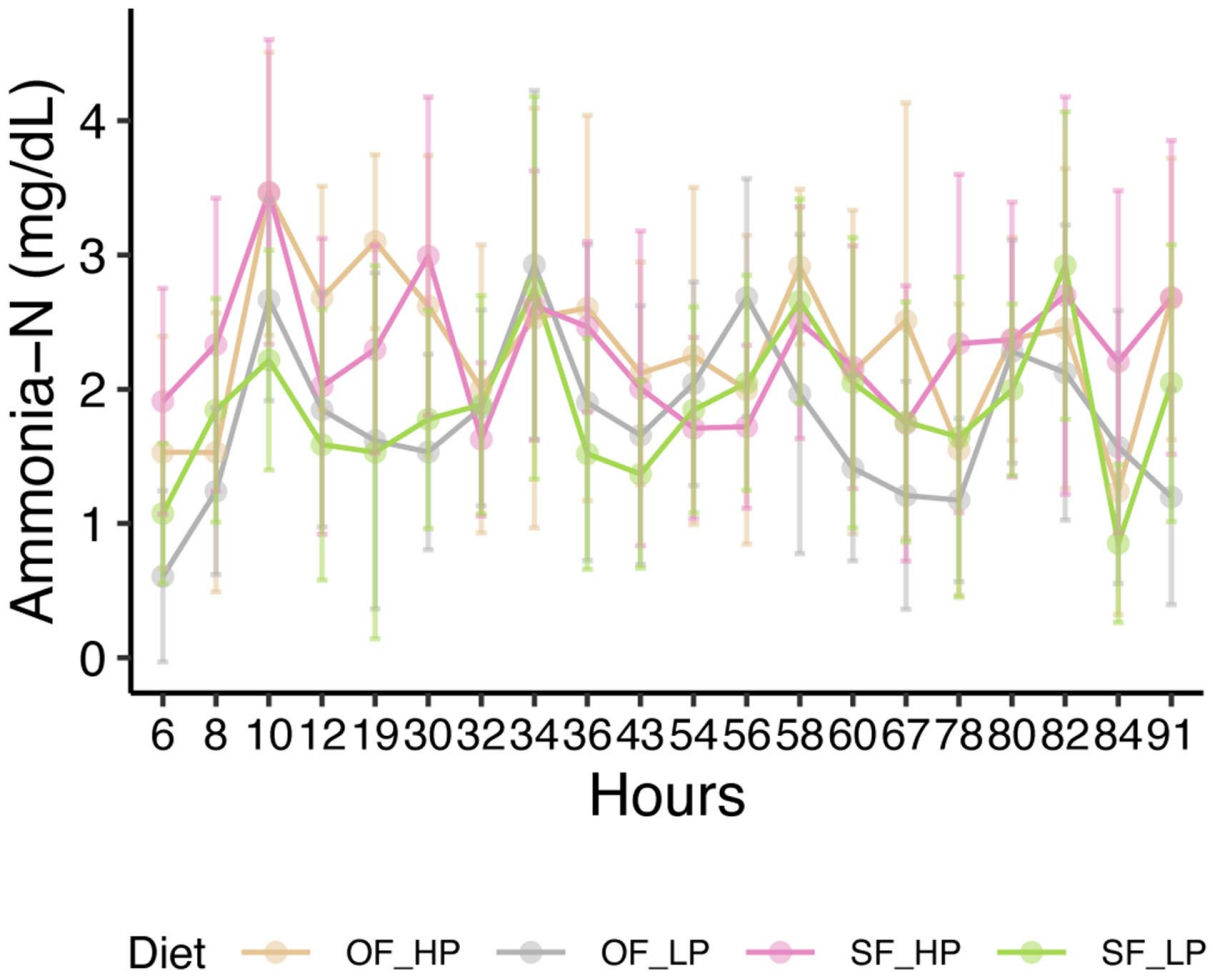
Ammonia nitrogen concentration in the rumen of dairy cattle subjected to diets with either high protein (HP) or low protein (LP) levels and oscillating feeding (OF) or static feeding (SF) patterns. Error bars represent the mean percentage of ammonia ± the standard deviation for each diet.

No differences were observed in the VFA profiles between feeding patterns or protein level (Table 1, Figure 1). Acetic acid was the most abundant VFA in the rumen (mean = 55.2 mM and SEM = 1.1 mM), followed by propionic acid (mean = 19.2 mM and SEM = 0.7 mM) and butyric acid (mean = 11.8 mM and SEM = 0.5 mM). No differences were observed in pH or total amino acids (Table 1). However, ammonia-N concentration varied significantly between low and high-protein diets (*p*-value under 0.001) (Table 1, Supplementary Figure 3). This difference in ammonia-N concentration persisted regardless of feeding patterns, and the variation of ammonia-N through time did not vary depending on the diet (Figure 2).

**Table 1:** Rumen fluid analyte least squares means, standard errors, and *p*-values for *F*-tests of treatment effects across days 25–28 of each period (*n* = 8 cows).

CP Level	LP		HP		SEM	*p*-values		
CP Feeding Pattern	OF	SF	OF	SF		CP level	CP feeding pattern	Interaction
pH	6.35	6.38	6.37	6.38	0.04	0.72	0.41	0.62
N components								
NH_3_–N (mg/dL)	1.76	1.92	2.30	2.35	0.14	<0.001	0.29	0.57
Total AA (mM)	2.70	2.82	2.56	2.50	0.2	0.25	0.88	0.65
VFA concentration (mM)								
Acetate	55.8	54.7	55.6	54.8	1.1	0.99	0.38	0.92
Propionate	19.0	18.6	19.5	19.8	0.7	0.21	0.93	0.55
Butyrate	11.7	11.9	11.8	11.8	0.5	0.88	0.79	0.74
Major VFA	86.4	85.2	86.9	86.4	1.7	0.63	0.63	0.82
Iso-valerate	1.27	1.38	1.35	1.52	0.08	0.14	0.07	0.64
Iso-butyrate	0.54	0.53	0.57	0.60	0.04	0.21	0.79	0.50
Total iso-acid	1.81	1.91	1.91	2.12	0.10	0.14	0.14	0.57

### 3.2 Sequencing and filtering

Using the filter parameters described above for the DADA2 algorithm, we recovered 4,733,651 sequences from the initial 5,194,880 raw sequences. The resulting amplicon size ranged from 220 and 339 bp, with a mean length of 252.76 bp. At the 9th percentile, the sequence length was 252 bp, and at the 98th percentile, it was 254 bp. We identified 17,886 ASVs. After removing ASVs classified as chloroplasts and mitochondria, the ASV count was reduced to 17,828. Finally, we used the decontam tool to filter out contaminants, resulting in a final ASV count of 17,808.

### 3.3 Diversity of microbial communities

#### 3.3.1 Alpha diversity

**Figure 3:**
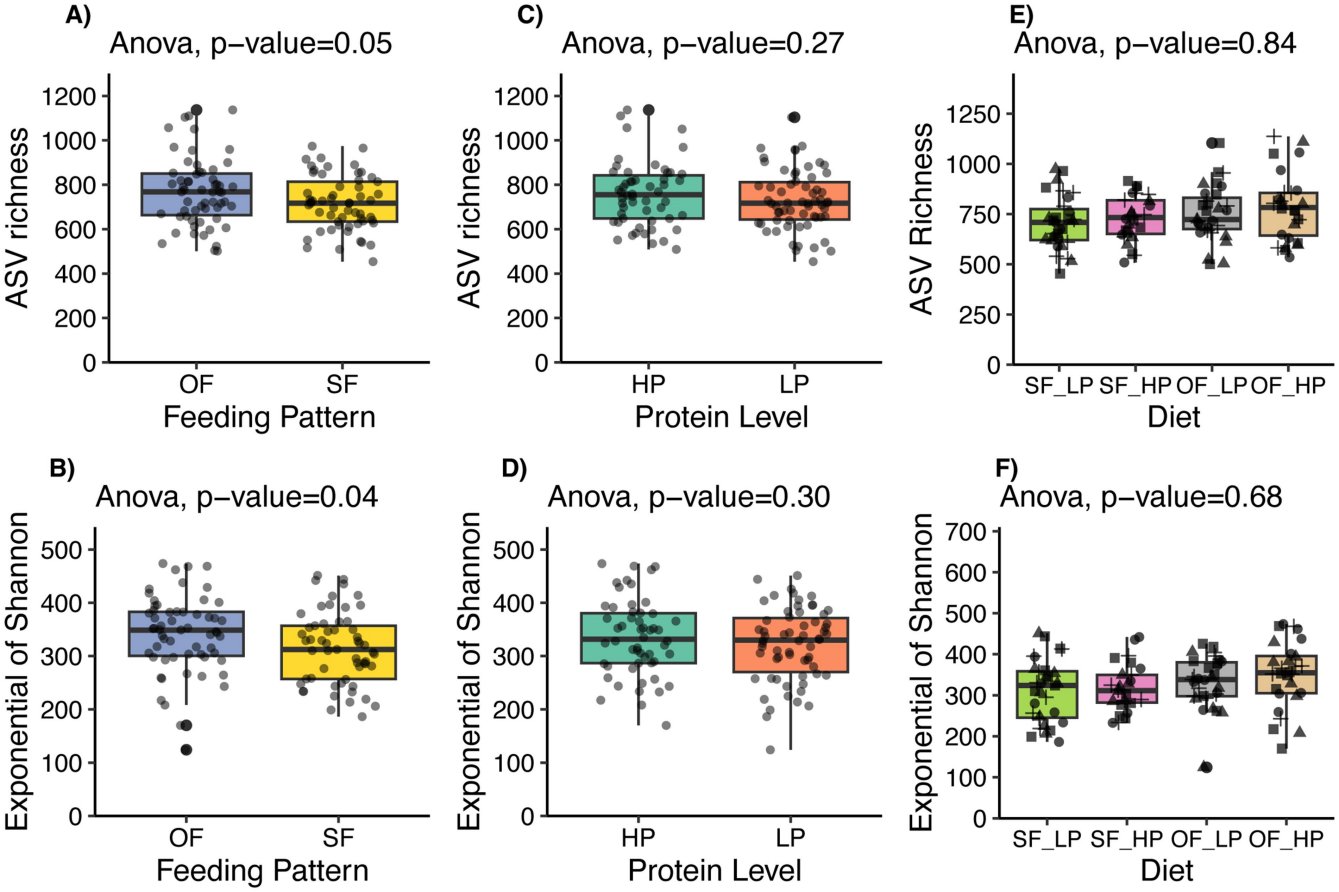
Alpha diversity indices of microbial communities in lactating dairy cattle subjected to oscillating dietary CP feeding (OF) or static feeding (SF) patterns, and high protein (HP) or low protein (LP) concentrations. Panels **(A,B)** display ASV richness and the Exponential of Shannon’s index, respectively, for both OF and SF patterns. Panels **(C,D)** show the same indices for LP and HP diets. Lastly, panels E and F show the effect of the diet or interaction between the feeding pattern and the protein level. Vertical bars indicate the standard deviation of the means.

Oscillating dietary CP levels led to some notable changes in the diversity of the ruminal microbiome of lactating Holstein cows. Specifically, cows subjected to an oscillating feeding pattern exhibited a tendency for increased microbial richness (*p* = 0.05), as indicated by ASV richness, and a significant increase in diversity (*p* = 0.038) as measured by the Exponential of Shannon’s index (Figure 3). In contrast, the comparison between high and low protein concentrations did not reveal significant differences in microbial richness (*p* = 0.27) or diversity (*p* = 0.30). Also, no difference was detected in richness (*p* = 0.84) or diversity (*p* = 0.68) based on the interaction of protein level and feeding pattern.

Further analysis of the feeding pattern showed that the microbial diversity did not differ between high protein and low protein diets when data were divided by feeding pattern (Supplementary Figures 4A–D). However, when the data were stratified by protein level, the oscillating feeding pattern within the high protein diet subset exhibited a trend towards increased diversity (*p* = 0.096) (Supplementary Figure 4G).

#### 3.3.2 Beta diversity

**Figure 4:**
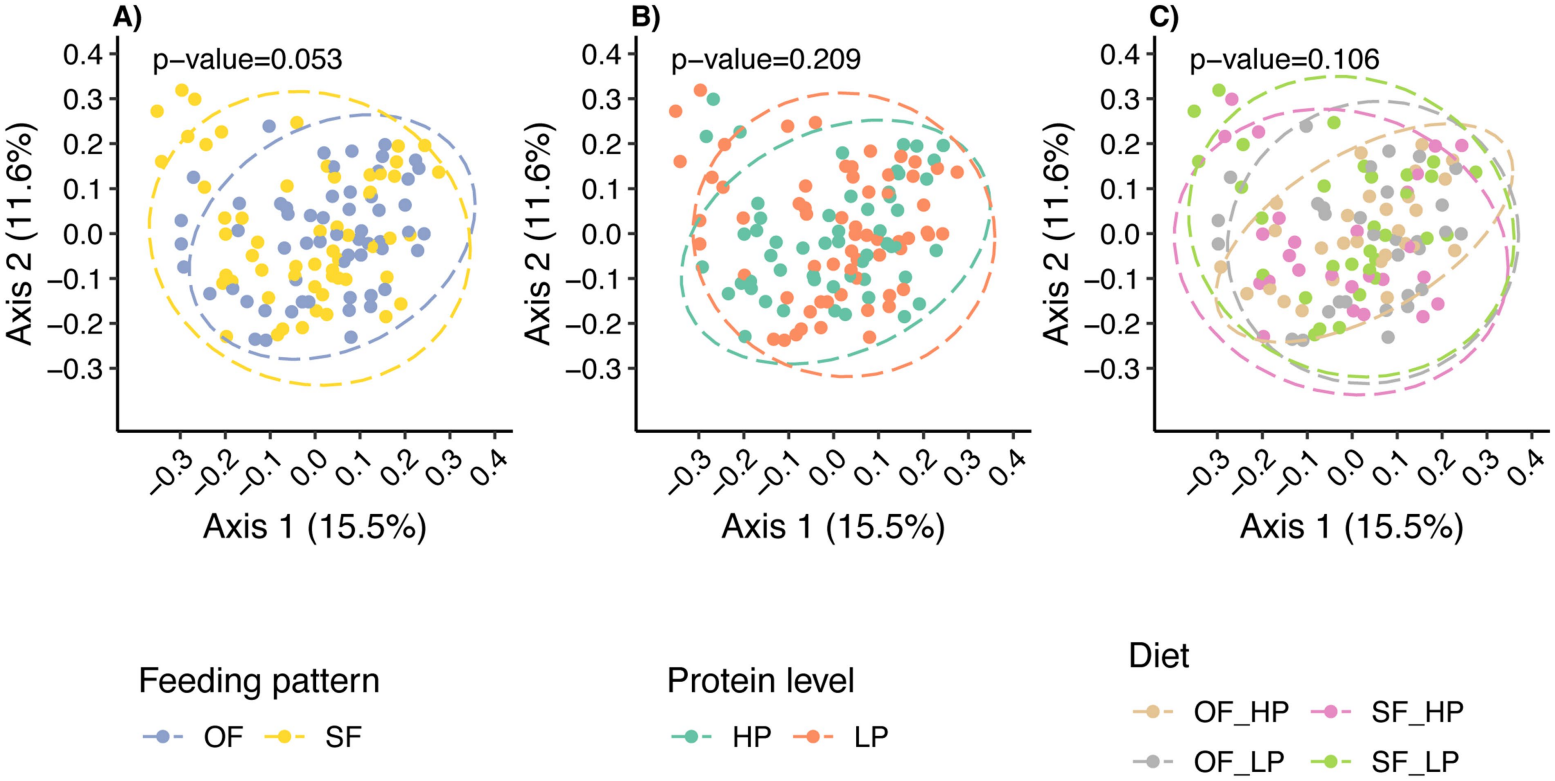
Principal Coordinates Analysis (PCoA) plots illustrating how feeding patterns and protein levels shaped the microbial community composition. In panel **(A)**, each dot represents a sample’s microbiome under either oscillating feeding (OF) or static feeding (SF) patterns, while panel **(B)** shows samples under high protein (HP) or low protein (LP) levels. Panel **(C)** shows the effect of the diet or interaction between the feeding pattern and the protein level. Each axis represents the percentage of variance explained by the Bray-Curtis distance, indicating the dissimilarity between microbiome compositions.

Beta diversity analysis revealed that the overall microbial composition was not significantly different among diets when assessed using the Bray-Curtis distance metric (Figure 4C). Nonetheless, there was a tendency for the feeding pattern to shift the microbial community composition (Figure 4A; *p* = 0.053), but no differences were observed in microbial composition when analyzed using weighted UniFrac distances, which considers evolutionary relationships between the species present in the microbial communities (Supplementary Figure 5A; *p* = 0.26). However, a significant difference was found using unweighted UniFrac, indicating a difference in community composition based on the presence or absence of taxa between feeding patterns (Supplementary Figure 5C; *p* = 0.031). Despite this, the low percentage of variance explained by the principal coordinate analysis (PCoA) axes suggests that these differences contribute minimally to the overall variability between treatments.

In contrast, protein levels did not significantly alter the microbial composition, as determined by Bray-Curtis (Figure 4B), weighted UniFrac (Supplementary Figure 5B), and unweighted UniFrac (Supplementary Figure 5D). This consistency aligns with the alpha diversity results, for which protein levels did not significantly impact microbial community diversity.

### 3.4 Taxonomic composition

**Figure 5:**
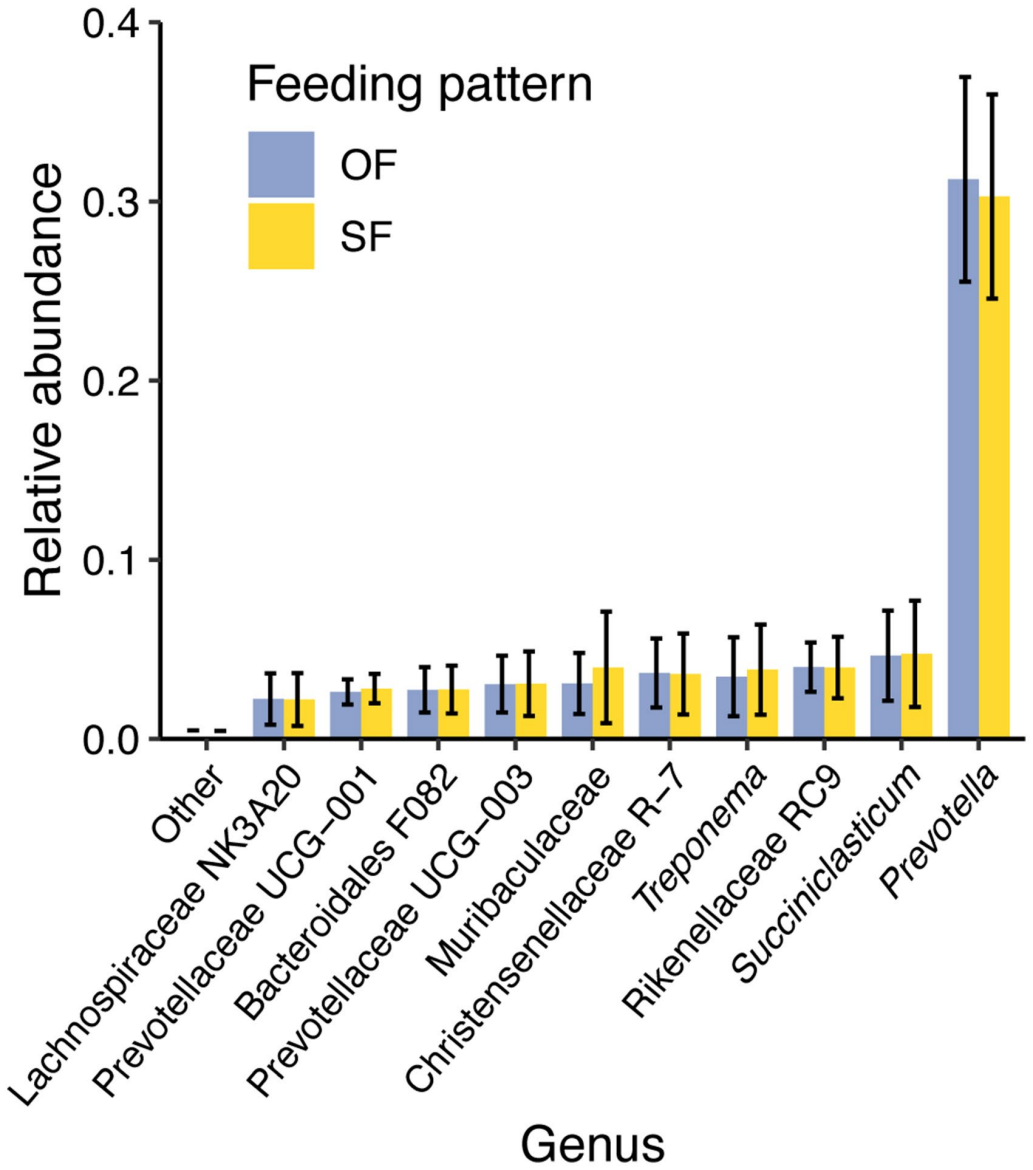
Mean relative abundance of the 10 most prevalent genera in the rumen microbiome of dairy cattle under oscillating feeding (OF) or static feeding (SF) patterns. Error bars represent the relative abundance ± the standard deviation for each feeding pattern.

Since a tendency was observed for feeding patterns to influence rumen microbial diversity, the relative abundances of the dominant taxa were compared between oscillating and static feeding patterns. *Prevotella* remained the predominant genus across all feeding patterns, with no significant differences in the relative abundance of 10 major taxa (Figure 5). The relative abundance of taxa not included in the major 10 was lower than 0.3% (Supplementary Table 2).

**Figure 6:**
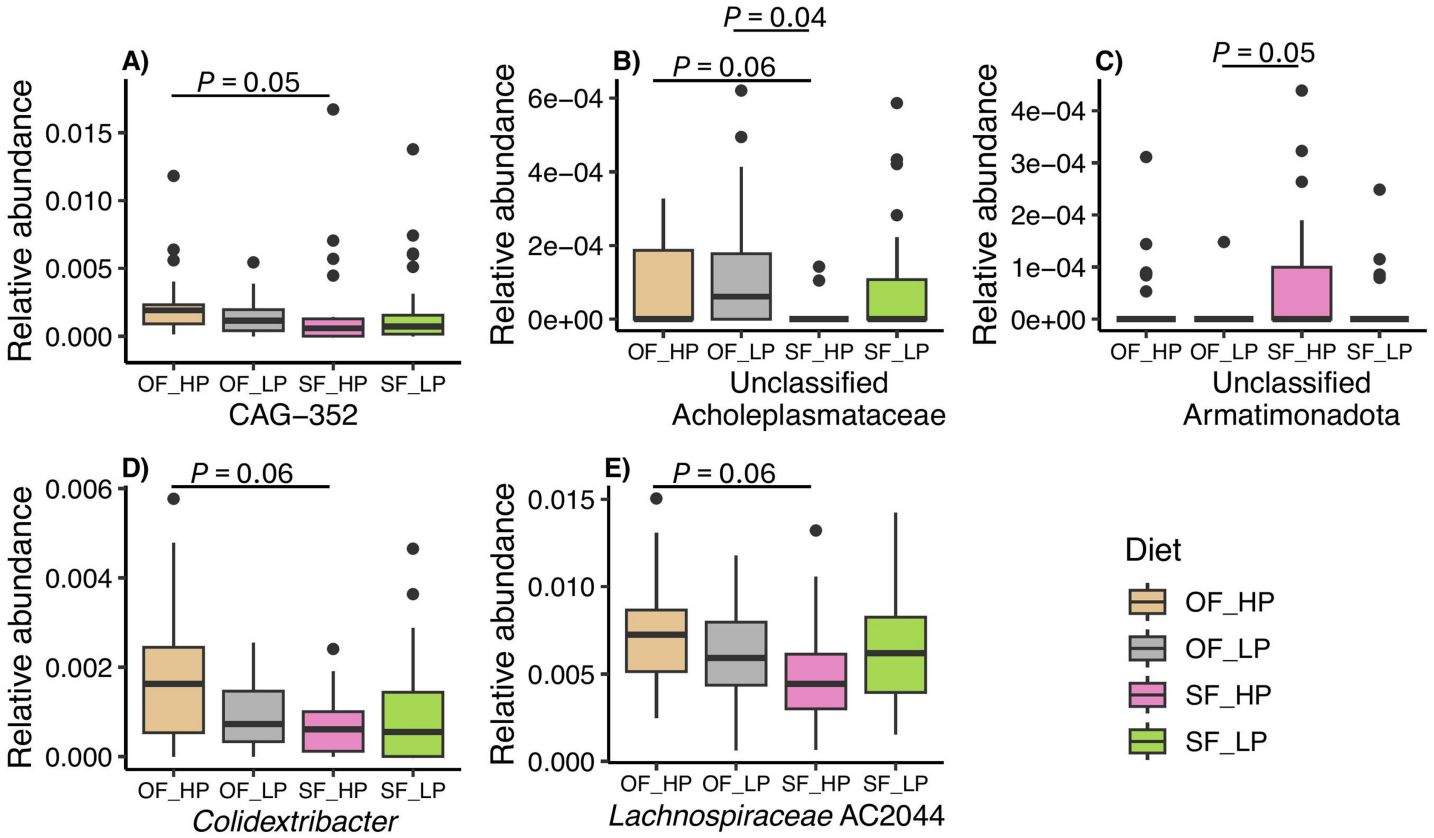
Differential abundant taxa in rumen microbiomes of dairy cattle subjected to diets with either high protein (HP) or low protein (LP) levels and oscillating feeding (OF) or static feeding (SF) patterns. Samples are grouped as OF-HP, OF-LP, SF-HP, or SF-LP. The y-axis displays the relative abundance (%) of ASVs classified within each differentially abundant genus. Each panel shows a different genus, panel **(A)** genus CAG-352, panel **(B)** unclassified genus of the family Acholeplasmataceae, panel **(C)** unclassified genus from the phylum Armatimonadota, panel **(D)** genus *Colidextribacter*, and panel **(E)** genus *Lachnospiraceae* AC2044.

We then sought to determine if there were any differentially abundant taxa between the oscillating and static feeding patterns or between the high and low protein levels used in our study. We found few taxa that were differentially abundant across all comparisons with the exception of the high dietary CP level. However, the relative abundance of these taxa was below 0.02% (Figure 6). For example, members of the genus CAG-352 (family Ruminococcaceae) were more abundant in the oscillating feeding pattern, when compared to the static pattern (Figure 6A, *p* = 0.05). A tendency was detected for the genera of the family Acholeplasmataceae with unclassified genus (Figure 6B, *p* = 0.06), *Colidextribacter* (Figure 6D, *p* = 0.06), and *Lachnospiraceae* AC2044 group (Figure 6E, *p* = 0.06), which were also more abundant in the oscillating diet of the high dietary CP level.

Accordingly, similar results were observed when the data were analyzed for the interaction between feeding pattern and protein level. Additionally, unclassified genera of the family Acholeplasmataceae (Figure 6B, *p* = 0.04) and the phylum Armatimonadota (Figure 6C, *p* = 0.05) were differentially abundant in the low protein-oscillating diet compared to the high protein-static diet. Finally, no differences were detected between static feeding diets. Nonetheless, in the global comparisons between taxa, the genera CAG-352 (*p* = 0.0001) and the genera from the family Acholeplasmataceae (*p* = 0.0002) were highlighted as differential.

### 3.5 Microbial function prediction

Regarding prediction accuracy, we evaluated the results of nearest-sequenced taxon index (NSTI) scores that summarize the extent to which microorganisms in a given sample are related to sequenced genomes (Douglas et al., 2020). A weighted NSTI score below 0.06 indicates high similarity to reference genomes, suggesting accurate predictions. Conversely, a score above 0.15 signifies low similarity and potential inaccuracies in the predicted taxonomic assignments. Also, NSTI per ASV > 2 is considered noise (Douglas et al., 2020). Our samples presented a weighed NSTI (mean ± SD) of 0.101 ± 0.009 while ASVs presented an NSTI of 0.256 ± 1.387 (see Supplementary Table 3). Approximately 105 ASVs presented NSTI score > 2 and were removed prior to downstream analysis.

**Figure 7:**
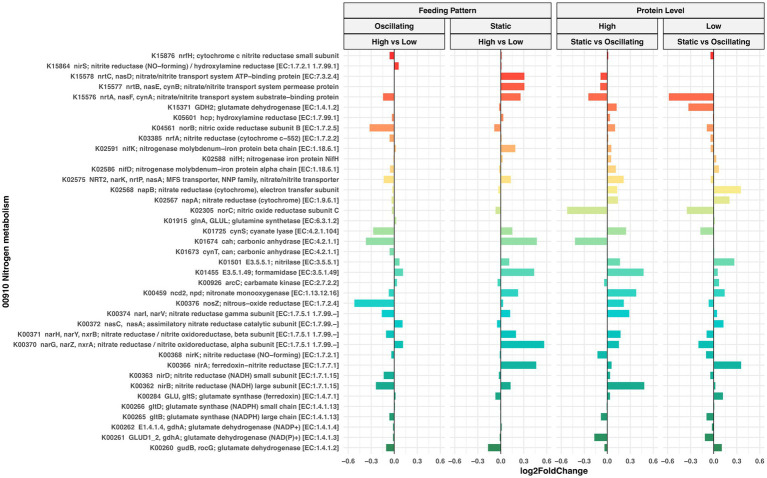
Differential abundance analysis of nitrogen metabolism orthologs (KEGG database) predicted by Picrust2. Waterfall plot illustrates Log2 fold changes (X-axis) in the abundance of predicted KOs (Y-axis) in rumen bacterial communities of cows fed diets with oscillating or static feeding patterns at high or low protein concentrations. Positive values indicate an increase, while negative values indicate a decrease in the KO abundance. None of the observed Log2 fold changes in this plot were found to be statistically significant (LinDA model, FDR > 0.05).

Next, we evaluated changes in the predicted functional profile of 16S rRNA bacterial communities in response to protein levels and feeding patterns. PICRUSt2 predicted the abundance of 6,280 functional orthologs (KOs), of which 599 were associated with Nitrogen, Sulfur, Nucleotide, and Amino acid metabolism pathways and present in at least 80% of our samples (Supplementary Figures 6, 7). The differential abundance analysis revealed that Log2Foldchange in the abundance of these KOs was relatively small and not statistically significant (FDR > 0.05) in pairwise comparisons between and within feeding pattern and CP level (Figure 7; Supplementary Figure 8).

## 4 Discussion

This study provides critical insights into the resilience and adaptability of the rumen microbiome in response to varying dietary CP levels, particularly under oscillating feeding patterns. Our primary finding that microbial function and milk production were maintained, even when dietary CP was reduced to 13.8% (Erickson et al., 2023), challenges the prevailing assumption that higher CP levels necessarily lead to higher dairy production (National Academies of Sciences, Engineering and Medicine, 2021). The overall stability in microbial composition and VFA concentrations observed in this study demonstrates the ability of the rumen microbiome to maintain a stable state after disturbance (varying dietary CP levels) and corroborates earlier findings indicating no significant changes in ruminal microbial communities of animals fed diets with differing N supplementation levels (Lopes et al., 2020).

The difference in dietary crude protein levels (13.8% vs. 15.5% CP) resulted in low ruminal ammonia-N concentrations among treatments. This likely reflects a combination of factors in the experimental design, including the relatively narrow range (1.7%) of rumen degradable and metabolizable protein between the two CP levels and the presence of rapidly fermentable carbohydrates from dietary starch, which can promote microbial assimilation of ammonia. While previous studies have demonstrated that reductions in dietary CP, high starch inclusion, or monensin supplementation can independently lower ruminal ammonia-N (Recktenwald et al., 2014; Belanche et al., 2012), our findings indicate that within a practical dietary range, these responses may converge to suppress ammonia-N concentrations. Notably, the feeding pattern (oscillating vs. static CP) had no significant effect on ammonia-N levels, suggesting that the rumen microbiota and host nitrogen recycling mechanisms help maintain stable nitrogen availability over time, even when dietary protein supply fluctuates from day to day. These results contrast with earlier reports of elevated ammonia-N under oscillating CP regimens (Köhler, 2016), highlighting the importance of contextual factors such as diet composition and protein oscillation amplitude.

Similarly, volatile fatty acid profiles were also unaffected by either CP level or feeding pattern, with acetate, propionate, and butyrate concentrations remaining stable. This aligns with studies reporting no or modest responses in VFA concentrations to changes in dietary protein within moderate ranges (Aguerre et al., 2016; Colmenero and Broderick, 2006) or oscillated dietary CP levels (Amaral et al., 2016; Köhler, 2016). Although we did not detect changes in branched-chain VFA concentrations, which are derived from amino acid fermentation, it is possible that the extent of proteolytic fermentation was limited by the relatively narrow variation in CP supply. In contrast, a previous study showing shifts in iso-acids (i.e., iso-butyrate and iso-valerate) involved more pronounced differences in dietary CP levels when using a dual-flow continuous culture system (Kidane et al., 2018). Overall, the ruminal fermentation profile observed in this study reflects a microbial ecosystem that maintains metabolic stability under dietary alterations in protein supply and feeding dynamics. These results support the interpretation that, under common dairy management practices, relatively small variations in CP content and feeding pattern are unlikely to meaningfully perturb core fermentation pathways.

Results also indicated no major shifts in bacterial richness or overall community structure across dietary protein levels. This apparent microbial stability may reflect the functional resilience of the rumen ecosystem rather than a lack of response. The few microbial taxa showing significant differential abundance were found in cows fed high dietary CP levels and were typically associated with rare taxa (abundance below 0.02%). Therefore, despite the effect detected on richness indices, the biological relevance of such modest changes in the overall community is probably limited. Importantly, the variation of crude protein tested (13.8% vs. 15.5% of DM) and the presence of sufficient fermentable energy likely contributed to maintaining microbial activity despite changes in nitrogen supply. These results contrast with findings from Parra et al. (2022), who reported increased bacterial richness and altered microbial profiles under low-protein diets in Brahman steers. However, such discrepancies are likely influenced by differences in host species, diet composition (CP levels varying from 8.8 to 13.5%), and experimental scale. While our study involved multiparous Holstein cows fed corn silage-and alfalfa-based diets, Parra et al. used a larger cohort of Brahman steers (*n* = 90) fed Rhodes grass with concentrate. Furthermore, in the current study, solid and liquid samples were combined to assess the entirety of the ruminal microbiome, which may have reduced the resolution of niche-specific microbial shifts. However, we did not expect or anticipate that modulating dietary crude protein would significantly impact the ruminal solid microbiome population (de Mendonça Lopes et al., 2023), and any differences in the abundances of known solid or liquid-associated ASVs would be readily identified in a comparison between samples, given the level of sequencing performed. The minimal response observed in the microbiome also aligns with the rumen fermentation results, particularly the stability in ruminal ammonia and VFA profiles. Taken together, these results suggest that the rumen microbiota may possess adaptive mechanisms, probably supported by microbial cross-feeding and host nitrogen recycling, that sustain core microbial functions and nitrogen utilization efficiency under modest variations in protein supply, even when extracellular ammonia concentrations are low (Stefański et al., 2020). The potential to reduce dietary CP levels without compromising production efficiency has far-reaching implications for dairy nutrition and environmental sustainability. Excess dietary CP can lead to increased N excretion, which poses environmental challenges, particularly in terms of urea-N excretion that may contribute to water pollution and manure ammonia emissions that contribute to air pollution (Pacheco and Waghorn, 2008). Furthermore, if the rumen microbiome operates most efficiently at a lower CP threshold, as suggested by our findings, this approach could prioritize N utilization efficiency and microbial protein production, leading to more precise and sustainable feeding practices compared with current practices aiming at meeting a benchmark CP level (typically with an additional “safety” margins) increasing the risk of overfeeding CP. Future studies using a broader range of protein levels and dietary conditions may be needed to detect more specific microbial responses.

An increase in microbial diversity was only observed under oscillating CP feeding patterns. As noted above, despite tendencies for shifts in beta diversity related to feeding patterns, no significant impact was observed on the fermentation process, as indicated by the stable proportion of major VFAs and the relative abundance of dominant microbial taxa. Moreover, the change in microbial diversity was not linked to significant changes in beta diversity or abundant taxa, which might indicate minimal biological relevance to ecosystem function. Furthermore, although a higher microbial diversity is often perceived as beneficial in many ecological contexts (Falentin et al., 2016; Ma et al., 2020), the implications for the rumen environment are complex. For example, a previous study suggested that lower microbial diversity might be associated with more efficient energy harvest in high-performing dairy cows (Shabat et al., 2016). Therefore, it is essential to determine whether an increase in diversity contributes to or detracts from overall rumen efficiency. Overall, the lack of significant impact of dietary CP level on the rumen microbiome composition suggests that the temporal dynamics of nutrient supply might be a more critical driver of microbial community structure than the absolute average concentration of CP within the tested range.

The oscillating feeding pattern also led to an increased abundance of lesser-known microbial taxa such as the candidate genus CAG-352. This genus has been reported previously in other ruminant studies, where it is positively correlated to various metabolic markers, including serum total protein, albumin, creatinine, and non-esterified fatty acids (Qiu et al., 2022). However, the functional role of CAG-352 within the rumen microbiome remains unclear (Jiao et al., 2022). Therefore, considering the lack of information about this taxon and its low relative abundance in samples from the current study, it is unclear whether it has a significant impact on the rumen ecosystem under these dietary conditions. Future research should focus on elucidating the ecological and metabolic contributions of these understudied taxa, particularly in the context of N utilization and protein metabolism.

Although no significant differences were detected in the potential function of the microbiome, the dietary conditions tested might promote different metabolic functions that could only be detected by a direct approach, such as transcriptomic analysis, which accounts for the actual gene expression or activity of microbial taxa. One limitation of functional inferences based on 16S rRNA gene data is the assumption that closely related organisms have similar genomic content and metabolic potential, which may not always hold true, particularly in complex ecosystems like the rumen, where strain-level variation is common. We observed that under static CP feeding conditions, low protein levels result in a microbiome potentially enriched with genes related to N and sulfur metabolism compared to high-protein diets. The same was true for the oscillation of protein levels compared to static feeding under high-protein diets. This supports Stefański et al. (2020) conclusion that the rumen microbiome transforms N more efficiently at lower concentrations of protein. Apparently, oscillation could also promote this efficiency, but these findings cannot be confirmed with our current approach.

Our study provides robust evidence for the general stability of the rumen microbiome composition under reduced CP levels. Moreover, we observed an increase in rumen ammonia levels in cows fed high CP diets, similar to findings reported by other authors (de Mendonça Lopes et al., 2023; Lopes et al., 2020). Lowering the dietary CP reduced the urine N excretion, which remained unaffected by the feeding pattern (Erickson et al., 2024). Altogether, these findings suggest lowering the dietary CP level will enhance N efficiency and reduce environmental impact. However, we must consider the limitations of this study, for example, to reach lower levels of dietary protein, dietary starch, and fiber were increased, and these factors might alter the parameters measured in the study.

Future studies should evaluate a broader range of protein levels in diets to identify the threshold at which rumen function is compromised. Additionally, identifying optimal microbial efficiency in terms of N utilization could lead to strategies focused on optimizing microbial protein production rather than merely achieving specific CP levels in the diet. This approach could enhance the sustainability and productivity of dairy operations, ultimately benefiting both industry and the environment.

## 5 Conclusion

This study demonstrates that variations in dietary crude protein (CP) concentration (13.8% vs. 15.5% of DM) and feeding pattern (static vs. oscillating) have limited effects on the composition and function of the rumen microbiome in lactating Holstein cows. Although oscillating CP feeding patterns increased microbial richness and diversity, these shifts were not associated with changes in dominant taxa or microbial function. Additionally, core fermentation parameters remained stable despite changes in CP supply, indicating strong functional resilience of the rumen microbial ecosystem. The lack of negative impacts on microbial activity or fermentation end-products when reducing dietary CP from 15.5 to 13.8% indicates an opportunity to improve nitrogen use efficiency without compromising rumen function. This has important implications for developing more sustainable protein feeding strategies in dairy production by lowering nitrogen excretion and reducing environmental footprint. Future research should evaluate broader dietary contrasts and integrate multi-omics approaches to uncover the metabolic consequences and long-term impacts of oscillating CP supply on host-microbiome interactions and production efficiency.

## Data Availability

The datasets presented in this study can be found in online repositories. The names of the repository/repositories and accession number(s) can be found below: https://www.ncbi.nlm.nih.gov/, PRJNA1215457.
