# Supplementary material for: Assessing the impact of oscillating dietary crude protein on the stability of the rumen microbiome in dairy cattle
Source: Front Microbiol. 2025 Jun 10;16:1568112. doi: 10.3389/fmicb.2025.1568112 (PMC12186453; doi:10.3389/fmicb.2025.1568112)
Supplement: Supplementary file 1 [file Supplementary_file_1.pdf]

# *Supplementary Material*

## 1 Supplementary Figures and Tables

### 1.1 Supplementary Figures

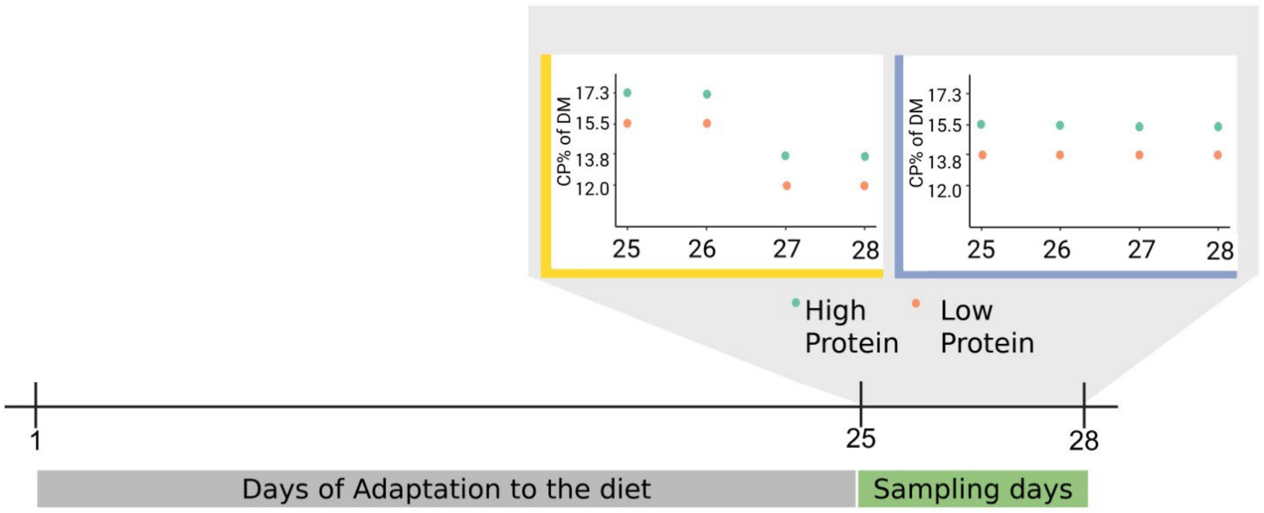


**Supplementary Figure 1.** Dietary crude protein concentration (CP, % of dry matter) in a 28-day experimental period with a 2x2 factorial design of CP level and feeding pattern. The CP level and CP feeding pattern combinations are oscillating low protein (OF-LP), oscillating high protein (OF-HP), static low protein (SF-LP), and static high protein (SF-HP). The sampling period (days 25 to 28) is highlighted, but the same diet was maintained throughout the diet adaptation period (days 1 to 24). Created in BioRender. Mantovani, H. (2025) https://BioRender.com/3p1tnrw

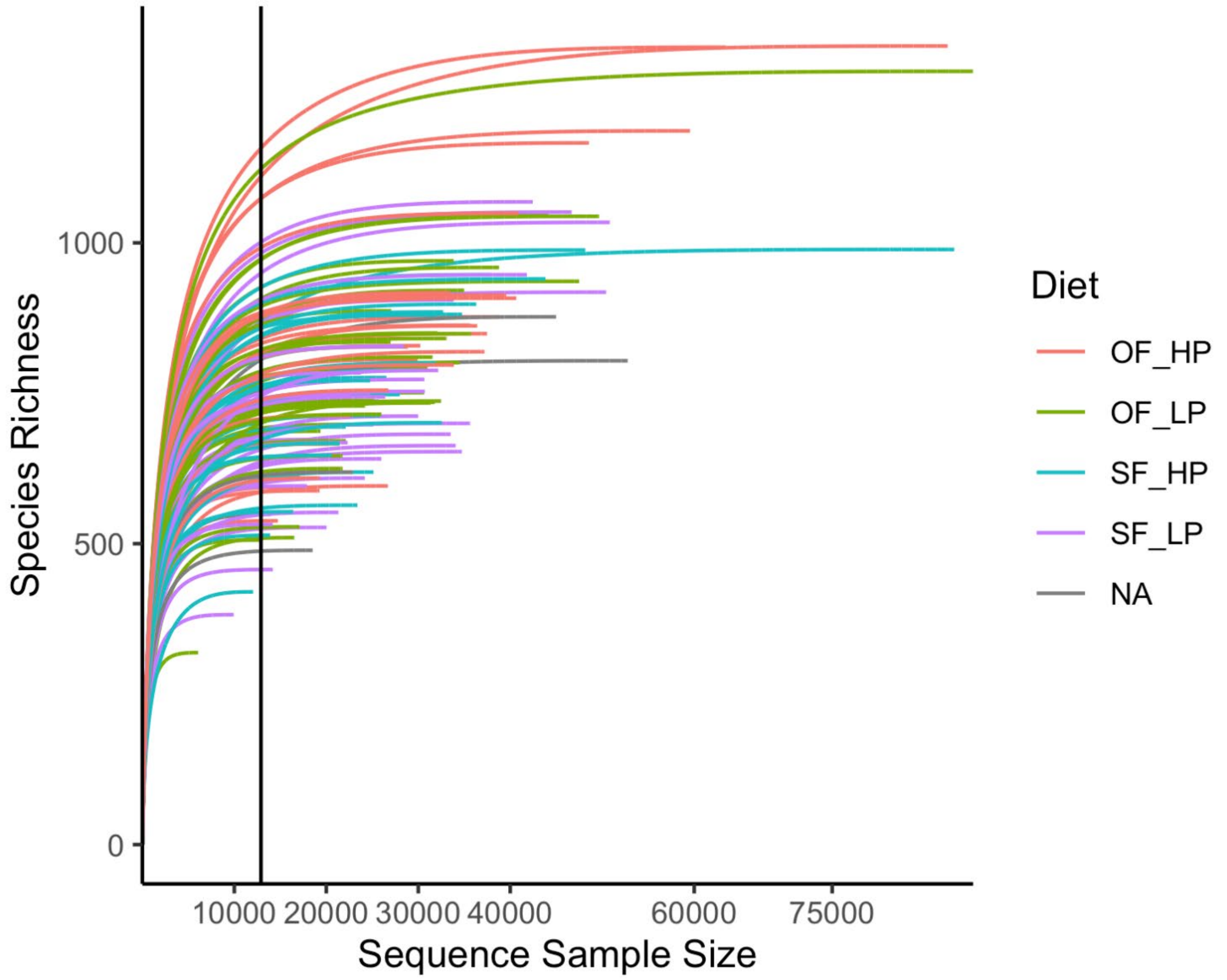


**Supplementary Figure 2.** Rarefaction curve: number of ASVs detected in each sample at different sampling depths.

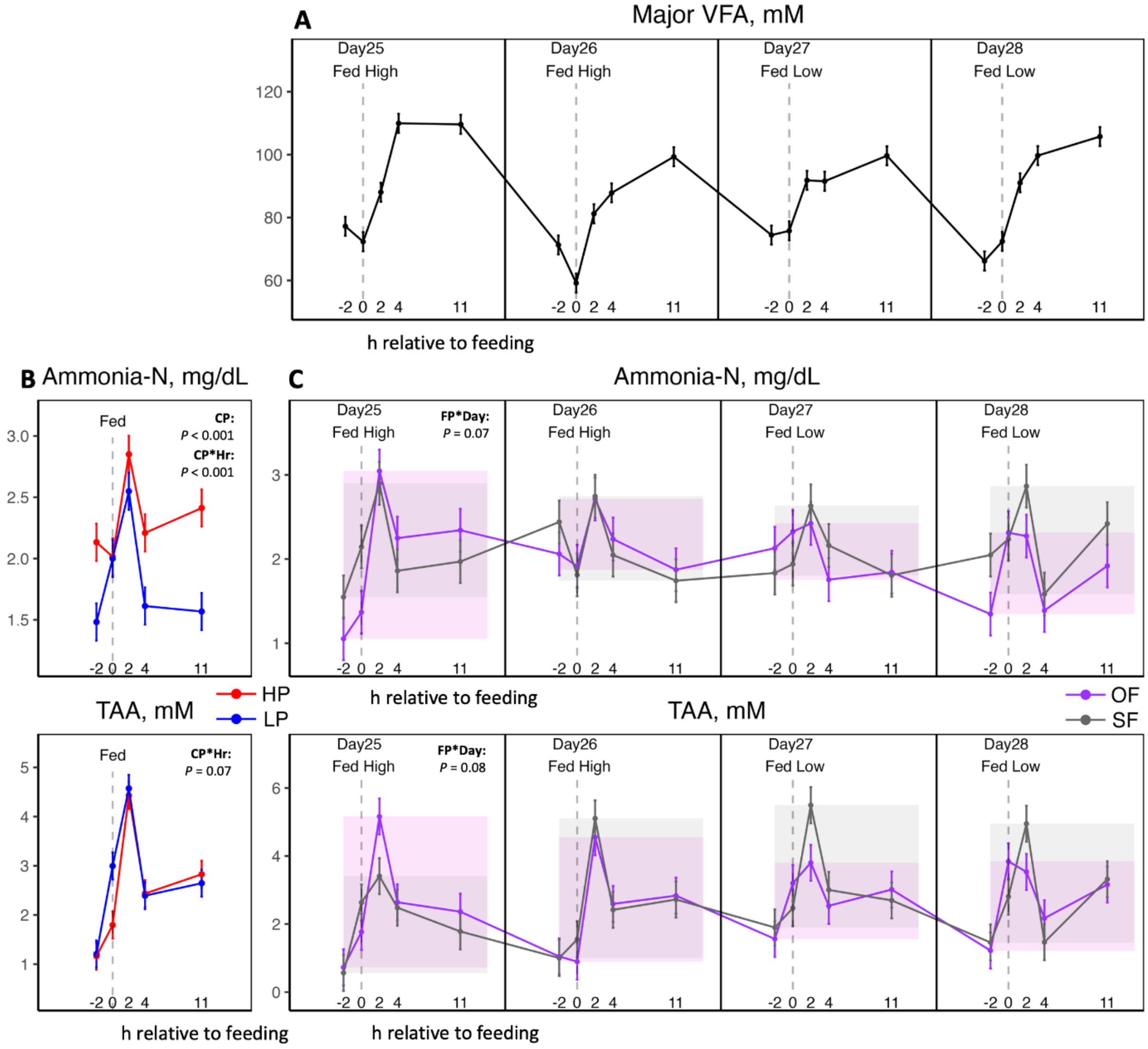


**Supplementary Figure 3.** Treatment and time effects on A) major VFAs concentration (sum of acetate, propionate, and butyrate), B/C) ammonia-N concentration, and C) total amino acid (TAA) concentration. Points and error bars show the least square means and standard errors for A) Day * Hr, B) CP level * Hr, and C) FP*Day*Hr, where shaded rectangles show the daily range of least squares means. Hr = Hour; CP = Crude protein; FP = Feeding pattern; * = interaction between variables.

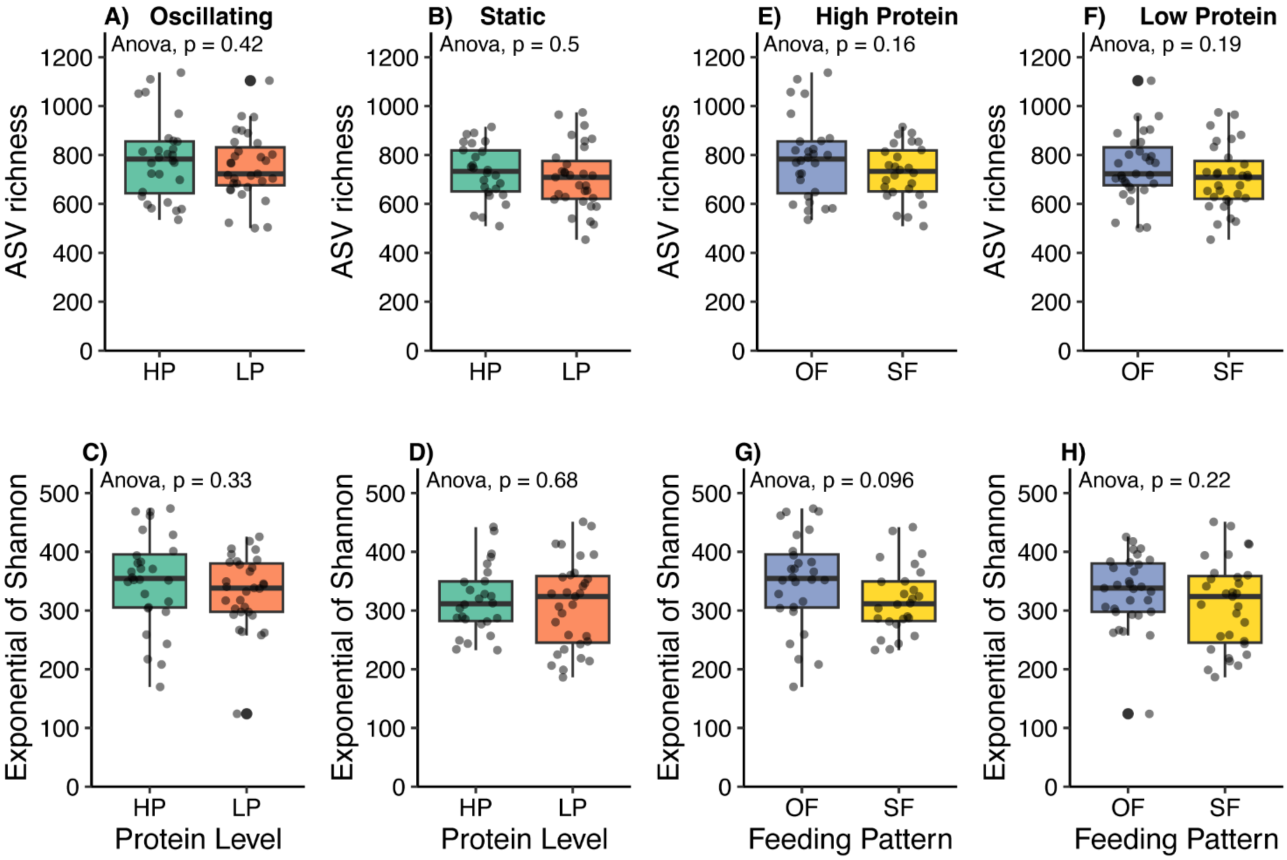


**Supplementary Figure 4.** Alpha diversity indices of rumen microbial communities in lactating dairy cows under oscillating feeding (OF) and static feeding (SF) patterns with high protein (HP) or low protein (LP) diets. ASV richness and the Exponential of Shannon's index are compared between HP and LP levels or OF and SF patterns. Panels A and C show samples from cows on OF patterns, while panels B and D show samples from cows on SF patterns. Panels E and G represent data for HP levels, and panels F and H for LP levels.

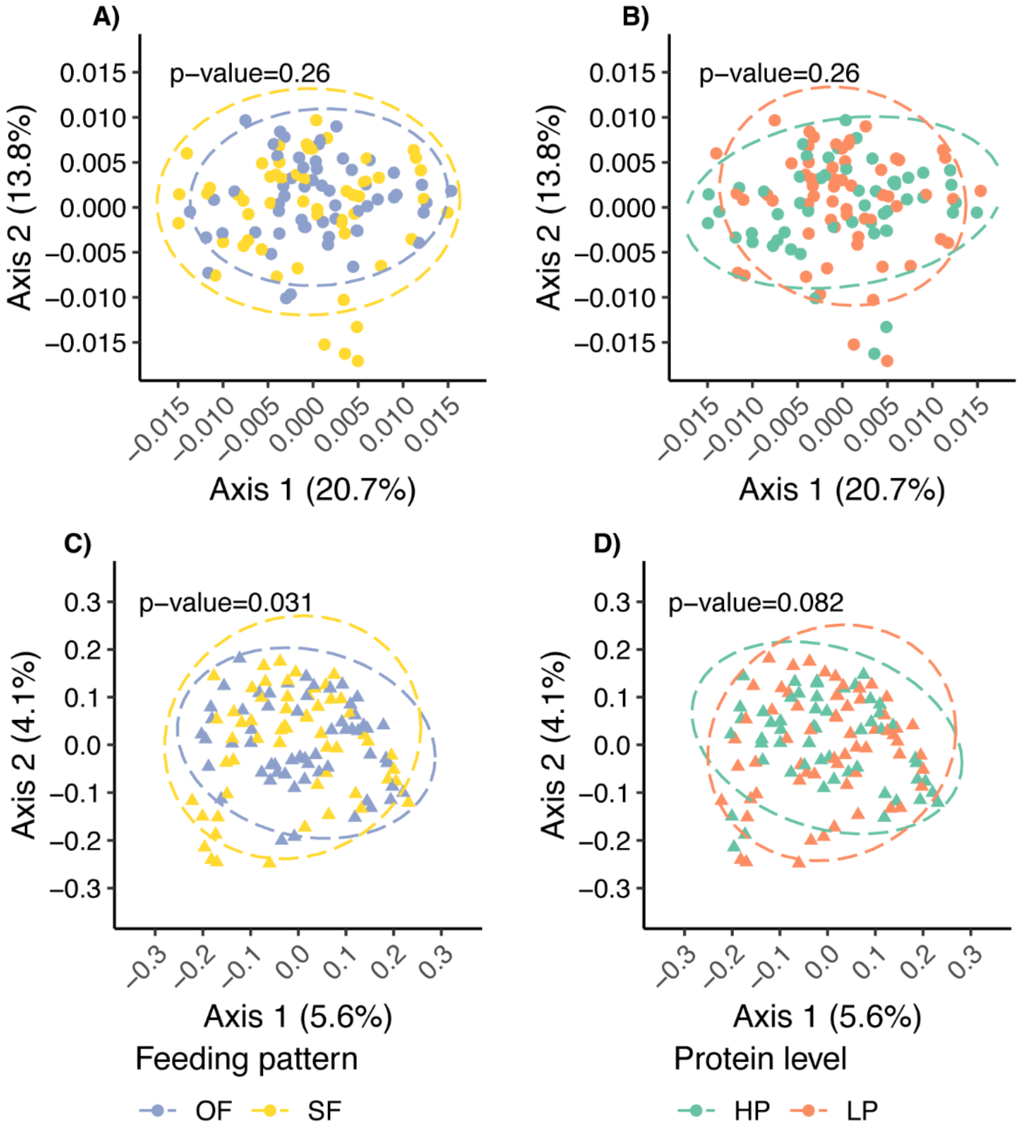


**Supplementary Figure 5.** Principal Coordinates Analysis (PCoA) plots showing microbial community structure, with each shape representing a sample's microbiome under oscillating feeding (OF) or static feeding (SF) patterns (Panels A and C) and high protein (HP) or low protein (LP) levels (Panels B and D). No significant differences were found between the ruminal microbial community composition of animals under OF and SF patterns or between HP and LP diets. Panels A and B represent weighted UniFrac distances among microbiomes, with each sample depicted as a circle. Panels C and D represent unweighted UniFrac distances, with each sample shown as a triangle. Each axis indicates the percentage of variance explained by the respective distance metrics.

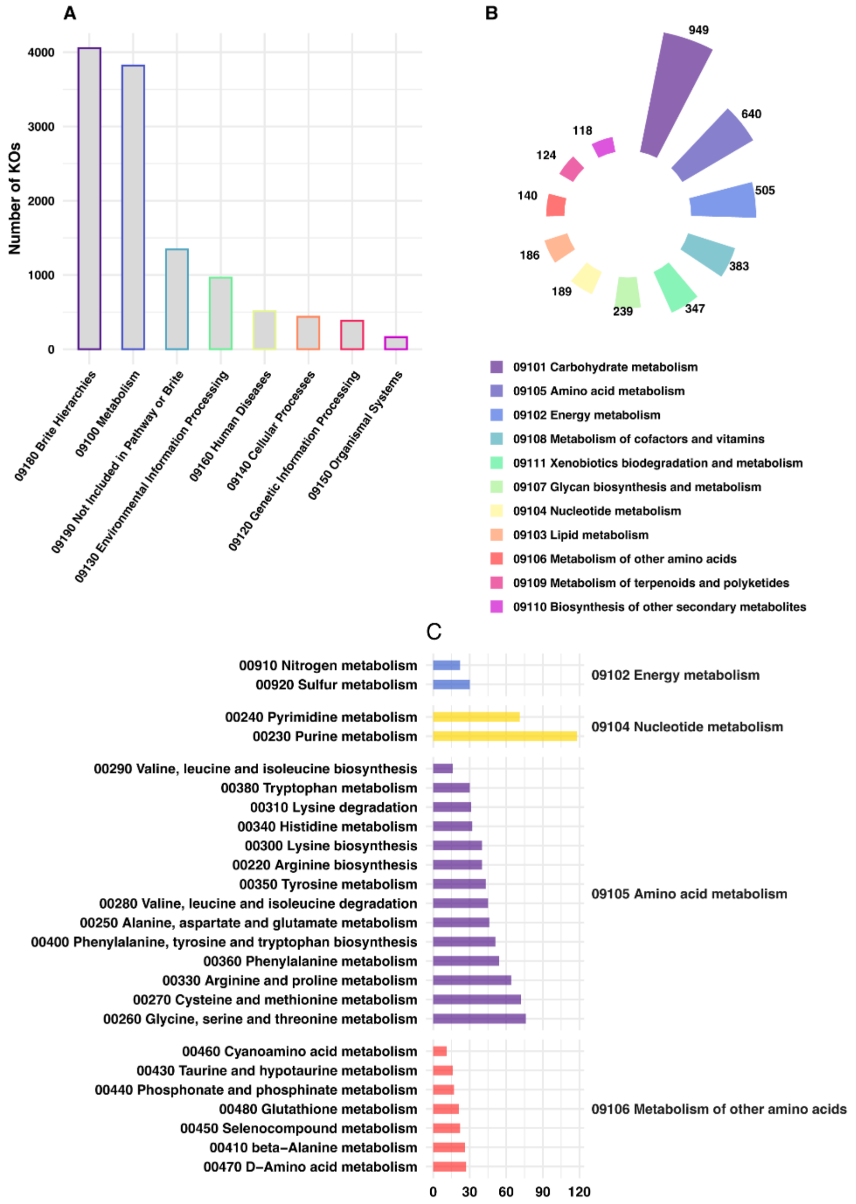


**Supplementary Figure 6.** Distribution of Picrust2 predicted functional orthologs (KO) of the KEGG database. Plots are showing the number of KOs per A) higher pathways nodes, B) secondary pathways nodes within 09100 Metabolism and C) per tertiary pathways nodes (09105 Amino Acid, 09102 Energy, 09104 Nucleotide metabolisms and 09106 Metabolism of other amino acids) that were selected for downstream analysis in our study.

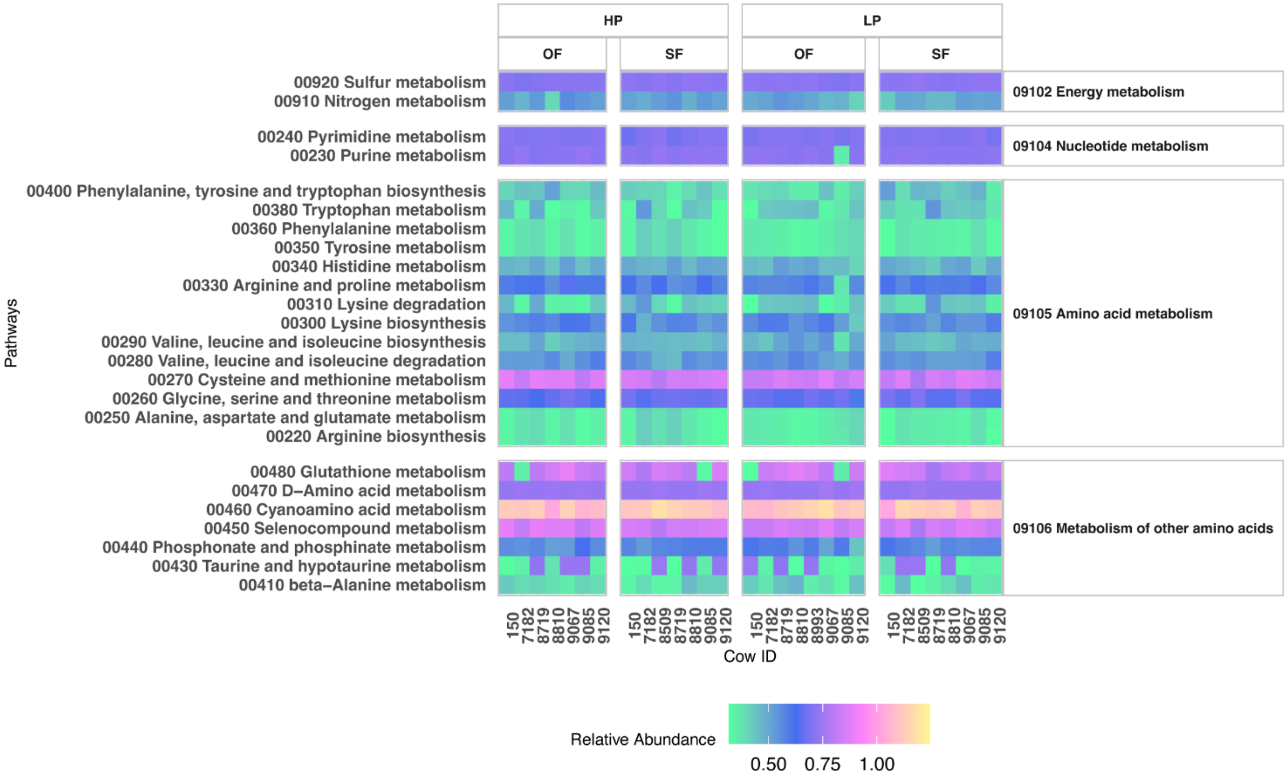


**Supplementary Figure 7.** Heatmap showing the relative abundance of the predicted functional orthologs (KO) summarized by higher pathways nodes (Y-axis) for each rumen sample/Cow (X-axis) fed diets with oscillating (OF) or static (SF) feeding patterns in high (HP) or low (LP) protein concentrations. The color key represents the relative abundance at gradient of color from green (low abundance) to gold (high abundance).

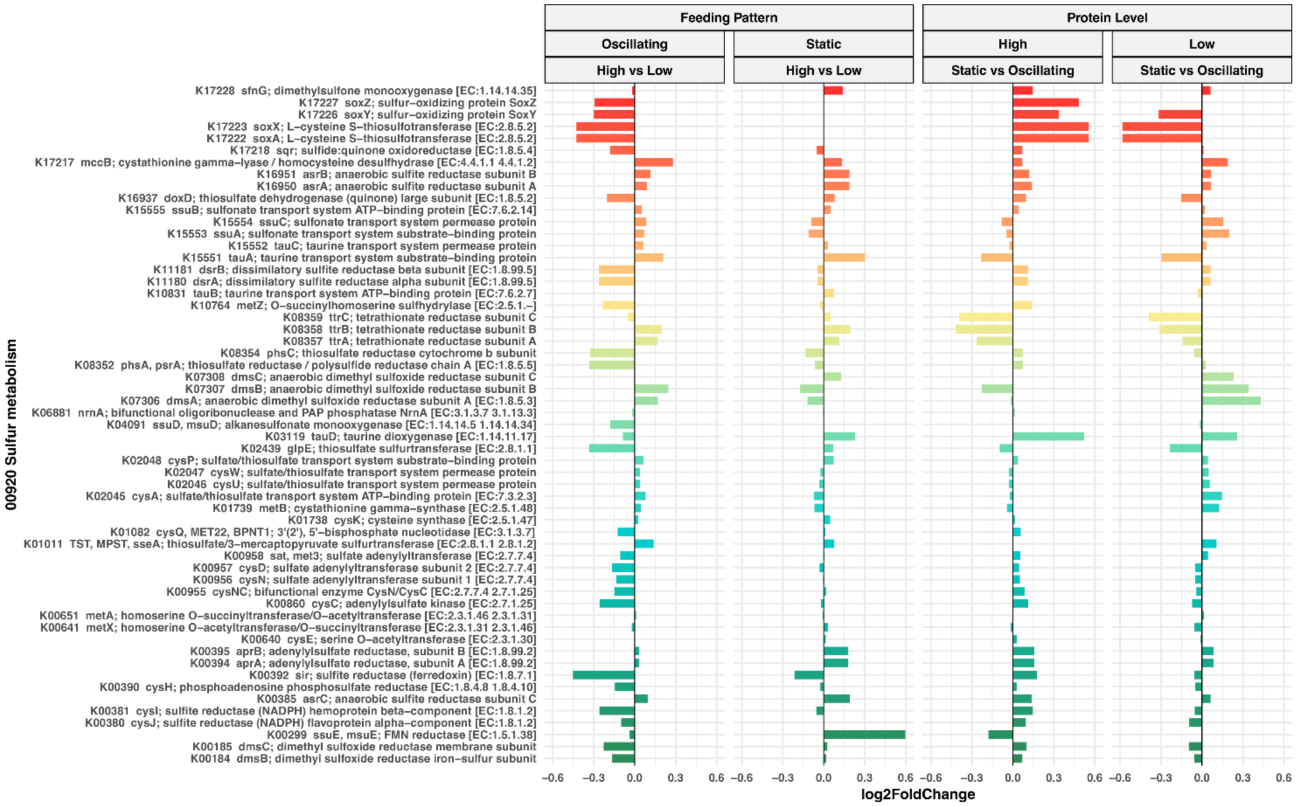


**Supplementary Figure 8.** Differential abundance analysis of sulfur metabolism orthologs (KEGG database) predicted by Picrust2. Waterfall plot illustrates Log2 fold changes (X-axis) in the abundance of predicted KOs (Y-axis) in rumen bacterial communities of cows fed diets with oscillating or static feeding patterns at high or low protein concentrations. Positive values indicate an increase, while negative values indicate a decrease in the KO abundance. None of the observed Log2 fold changes in this plot were found to be statistically significant (LinDA model, FDR>0.05).

**Sub. Table 1.** Comparison of statistical linear models.

| | | ASV richness | | | Exponential of Shannon | | |
|---|---|---|---|---|---|---|---|
| | Model | Adj. R | AICc | AICc Wt | Adj. R | AICc | AICc Wt |
| 1 | $\beta_0+\beta_1 PL_i+\beta_2 OS_i+\epsilon_i$ | 0.03 | 1477.18 | 0.68 | 0.03 | 1318.71 | 0.63 |
| 2 | $\beta_0+\beta_1 PL_i+\beta_2 OS_i+\beta_3 D_i+\epsilon_i$ | 0.02 | 1479.32 | 0.23 | 0.02 | 1320.72 | 0.23 |
| 3 | $\beta_0+\beta_1 PL_i+\beta_2 OS_i+\beta_3 D_i+\beta_4 C_i+\epsilon_i$ | 0.03 | 1481.98 | 0.06 | 0.08 | 1323.34 | 0.06 |
| 4 | $\beta_0+\beta_1 PL_i+\beta_2 OS_i+\beta_3 D_i+\beta_4(PL_i\times D_i)+\beta_5(OS_i\times D_i)+\epsilon_i$ | -0.01 | 1484.24 | 0.02 | 0.02 | 1323.73 | 0.05 |
| 5 | $\beta_0+\beta_1 PL_i+\beta_2 OS_i+\beta_3 C_i+\beta_4(PL_i\times C_i)+\beta_5(OS_i\times C_i)+\epsilon_i$ | 0.02 | 1487.66 | 0 | 0.03 | 1325.83 | 0.02 |
| 6 | $\beta_0+\beta_1 PL_i+\beta_2 OS_i+\beta_3 D_i+\beta_4(PL_i\times D_i)+\beta_5(OS_i\times D_i)+\beta_6 C_i+\beta_7(PL_i\times C_i)+\beta_8(OS_i\times C_i)+\epsilon_i$ | -0.02 | 1490.99 | 0 | 0.02 | 1327.42 | 0.01 |
| 7 | $\beta_0+\beta_1 PL_i+\beta_2 OS_i+\beta_3(PL_i\times OS_i)+\epsilon_i$ | 0.02 | 1492.77 | 0 | 0.02 | 1331.35 | 0 |
| 8 | $\beta_0+\beta_1 PL_i+\beta_2 OS_i+\beta_3 D_i+\beta_4(PL_i\times OS_i)+\epsilon_i$ | 0.01 | 1510.54 | 0 | 0.01 | 1350.88 | 0 |
| 9 | $\beta_0+\beta_1 PL_i+\beta_2 OS_i+\beta_3 C_i+\beta_4(PL_i\times OS_i)+\epsilon_i$ | 0.04 | 1535.24 | 0 | 0.09 | 1373.29 | 0 |

AICc: Akaike Information Criterion corrected

AICc Wt: Akaike Information Criterion corrected weight

*PL*: Dietary CP Level

*OS*: Dietary CP feeding pattern

*D*: Sampling Day

*C*: Cow

*P*: Period

$\epsilon_i$: residual

$\beta_0$: intercept of the model

$\beta_i$: coefficient of each variable

**Sup. Table 2.** Abundance and relative abundance of each taxon at the genus level quantified in each sample.

**Sup. Table 3.** Quality control of Picrust2 predictions: weighted nearest-sequenced taxon index (NSTI) per sample and summarized (mean ± standard deviation) per feeding pattern and protein level.

| **LP** | | **HP** | |
|---|---|---|---|
| **OF** | **SF** | **OF** | **SF** |
| 0.103 ± 0.007 | 0.100 ± 0.012 | 0.102 ± 0.009 | 0.102 ± 0.009 |
